# FGF2/FGFR1 regulates autophagy in FGFR1-amplified non-small cell lung cancer cells

**DOI:** 10.1186/s13046-017-0534-0

**Published:** 2017-05-30

**Authors:** Hong Yuan, Zi-Ming Li, Jiaxiang Shao, Wen-Xiang Ji, Weiliang Xia, Shun Lu

**Affiliations:** 1 Shanghai Lung Cancer Center, Shanghai Chest Hospital, Shanghai Jiao Tong University, Shanghai, 200030 China; 2 School of Biomedical Engineering and Med-X Research Institute, Shanghai Jiao Tong University, Shanghai, 200030 China

**Keywords:** NSCLC, FGFR1, ERK, Beclin-1, Autophagy

## Abstract

**Background:**

Autophagy is a conserved catabolic process to degrade cellular organelles. The role of autophagy in cancer development is complex. Amplification of fibroblast growth factor receptor 1 (FGFR1) is one of the most frequent targets in lung squamous cell carcinoma (SQCC). Whether fibroblast growth factor 2 (FGF2)/FGFR1 contributes to the regulation of autophagy remains elusive.

**Methods:**

Autophagic activity was evaluated by immunoblotting for microtubule-associated protein 1 light chain 3 (LC3), formation of GFP-LC3 puncta, and monodansylcadaverine (MDC) staining. The effect of autophagy inhibition on cell survival was assessed by cell viability and apoptosis assays.

**Results:**

We elucidated that FGFR1 activation suppressed autophagy. Pharmacological or genetic inhibition of FGFR1 by AZD4547 or FGFR1 short hairpin RNA (shRNA) induced autophagy in FGFR1-amplified non-small cell lung cancer (NSCLC) cells, H1581 and H520 cells. Mechanistic study revealed that the induction of autophagy by FGFR1 inhibition was mediated through inhibiting the ERK/MAPK pathway not by AKT pathway, accompanied by upregulation of beclin-1. Furthermore, activation of ERK/MAPK by transfection with a constitutively active MEK1 (caMEK1) construct or knockdown of beclin-1 by RNAi could attenuate autophagy induced by FGFR1 inhibition. Beclin-1 expression was inversely correlated with MEK1 phosphorylation. Inhibition of autophagy by beclin-1 silencing could enhance apoptosis after AZD4547 treatment in H1581 and H520 cells. High levels of LC3B mRNA was a marker of poor prognosis in NSCLC patients.

**Conclusions:**

Simultaneously inhibiting FGFR1 and autophagy could enhance cell death which should be further explored in vivo.

**Electronic supplementary material:**

The online version of this article (doi:10.1186/s13046-017-0534-0) contains supplementary material, which is available to authorized users.

## Background

Lung cancer is the leading cause of cancer-related mortality worldwide [1]. Lung squamous cell carcinoma (SQCC) comprises approximately 30% of non-small cell lung cancer (NSCLC). In contrast to the significant advances in lung adenocarcinoma, no therapeutically tractable targets have been identified in lung SQCC [2]. Patients with lung SQCC are currently treated with standard chemotherapy, which yields generally poor outcomes. Therefore, there is an urgent need to develop more efficacious targeted treatments.

Fibroblast growth factor receptor 1 (FGFR1), an oncogenic receptor tyrosine kinase (RTK), plays fundamental roles in physiological processes and cancer progression [3]. Under normal conditions, FGFR1 signaling is triggered by growth factors, such as fibroblast growth factor 2 (FGF2), leading to receptor dimerization and transphosphorylation of FGFR1. The activated FGFR1 results in the activation of downstream signaling pathways including the RAS/MAPK pathway, the PI3K/AKT signaling, and STAT-dependent pathway [3]. Aberrant FGFR signaling due to FGFR amplification, autocrine stimulation, gene fusion, or activating mutation is associated with many cancers [4]. The frequency of FGFR1 amplification is 21–22% in lung SQCC samples [5–8].

Macroautophagy (herein referred to as autophagy) is an evolutionarily conserved catabolic process for the degradation of various cellular constituents, such as long-lived proteins and cytoplasmic organelles [9, 10]. The beclin-1-interacting complex promotes autophagosomal membrane nucleation, whereas the Atg12-Atg5-Atg16 and microtubule-associated protein 1 light chain 3 (LC3)/Atg8 conjugation systems are important for autophagosomal elongation [11]. The role of autophagy in cancer development seems to be controversial. Some studies suggest that impaired autophagy may contribute to cancer development [12, 13]. Others show autophagy promotes cancer cell survival and is pro-tumorigenic [14, 15]. According to an emerging hypothesis, autophagy may enable tumor cells to evade treatments and induce therapeutic resistance [16]. Further, autophagy and apoptosis might be linked to each other and occur simultaneously or sequentially in a context dependent manner [17]. In NSCLC, there are only very few studies on the prognostic value of the expression of autophagy-associated markers, namely LC3A, LC3B, beclin-1, and SQSTM1 [18–20]. Therefore, it is urgent to investigate the impact of autophagy on tumorigenesis and chemosensitivity, especially in FGFR1-amplified lung SQCC.

Several studies have shown that FGFR signaling regulates autophagy. It was reported that the FGF signaling axis activated mTOR and suppressed autophagic activity via FGF receptor substrate 2α (FRS2α)-mediated PI3K/AKT pathway in mouse embryonic fibroblasts [21]. It has demonstrated FGF/FRS2α-mediated signals prevented premature differentiation of cardiac stem cells via inhibition of autophagy [22]. More recently, Wang et al. [23] have revealed that activation of FGFR3 inhibited autophagic activity through decreasing the level of ATG12-ATG5 conjugation in chondrocytes. Settembre et al. [24] have demonstrated that the growth factor FGF18 induced the activation of FGFR4 and JNK, which activated the autophagy initiation complex VPS34-beclin-1 during post-natal bone growth in chondrocytes. All these studies suggest that FGF signaling axis plays an important role in cell differentiation, heart development, and bone growth through regulating autophagy. However, the underlying molecular mechanisms by which FGF2/FGFR1 regulates autophagy in FGFR1-amplified NSCLC remain elusive.

In this study, we explored the effect of FGF signaling axis for regulation of autophagy in FGFR1-amplified NSCLC cells. Using in vitro cultured FGFR1-amplified NSCLC cell lines, we demonstrate that FGFR1 activation promotes autophagy inhibition. Suppression of FGFR1 increases the protein level of beclin-1 and induces autophagy through inhibiting the ERK/MAPK pathway. Genetic inhibition of autophagy enhances cell death of H1581 and H520 cells after AZD4547 treatment. Our findings provide a novel insight into the role of autophagy in FGFR1-amplified NSCLC and have important implications for autophagy inhibition in FGFR1-targeted therapy.

## Methods

### Reagents and antibodies

Recombinant human FGF2 was purchased from Invitrogen. A selective inhibitor of FGFR1, 2 and 3, AZD4547 (a gift from AstraZeneca) was dissolved in dimethyl sulfoxide (DMSO) for storage [25]. E-64d, pepstatin A and monodansylcadaverine (MDC) were purchased from Sigma-Aldrich Corp. Z-VAD-fmk was obtained from Gene Operation. The antibodies for western blotting and their relevant sources are as follows: Anti-LC3B (Sigma-Aldrich); GAPDH (Abmart); Tubulin (Abcam); total and Tyr653/654-phosphorylated FGFR, total and T202/Y204-phosphorylated extracellular signal-activated kinase (ERK), total and Tyr705-phosphorylated Stat3, PARP and Caspase-9 (Cell Signaling Technology, Inc.); total and S473-phosphorylated AKT1 (Epitomics); beclin-1 (Proteintech). Secondary antibodies were peroxidase-conjugated AffiniPure Goat anti-mouse IgG and anti-rabbit IgG (Jackson ImmunoResearch Laboratories, INC.).

### Cell lines and cultures

The NSCLC cell lines, H1581 and H520 were purchased from the American Type Culture Collection (ATCC). SK-MES-1 cells were kindly provided by Cell Bank, Chinese Academy of Sciences, Shanghai Branch (Shanghai, China). H1581 and H520 cells were grown in RPMI-1640 medium (Hyclone) supplemented with 10% fetal bovine serum (FBS) (Invitrogen), 100 U/ml penicillin and 100 μg/ml streptomycin (Invitrogen), and incubated in a humidified atmosphere (95% air and 5% CO_2_) at 37 °C. SK-MES-1 cells were cultured in MEM medium (Hyclone) with 10% FBS (Invitrogen), 100 U/ml penicillin and 100 μg/ml streptomycin. All cells were passaged fewer than 6 months after receipt from cell bank or resuscitation.

### Western blotting

Cultured cells were harvested with a rubber scraper and washed twice with cold phosphate buffered saline (PBS). Cell pellets were lysed and kept on ice for at least 20 min in RIPA lysis buffer (Millipore), with addition of phenylmethylsulfonyl fluoride and protease inhibitors cocktail (Thermo Scientific). The lysates were cleared by centrifugation, and the supernatants were collected. Proteins were quantified by the BCA assay, and loading buffer 5X was added to the proteins which were incubated for 5 min at 95 °C. Then, proteins were loaded on SDS-PAGE poly-acrylamide gel, transferred to Immobilon-P PVDF membrane (Millipore), probed with the appropriate primary antibodies, and detected by chemoluminescence (ECL, Thermo Scientific). Images were then acquired with an ImageLab software (BioRad). Image analysis of western blots was performed with Gel-Pro analyzer software.

### Immunofluorescence Microscopy

Cells were plated on glass chambers (NEST) at 60% confluency and incubated in normal medium or serum-free medium for overnight. H1581 and H520 cells were then either untreated or stimulated with FGF2 (25 ng/ml) for 2 h. Cells were washed in PBS, fixed with 4% paraformaldehyde (PFA) for 15 min at room temperature (RT). Coverslips were mounted on microscope chamber slides with DAPI (Beyotime) for 5 min at RT. Representative images were chosen and exported from the Leica Application Suite software using identical settings for each set of control versus experimental conditions.

### Autophagy Assays

Autophagic activity was analyzed by detecting the LC3-II turnover, GFP-LC3 puncta, and MDC staining [26–28]. For LC3-II turnover, 15% of SDS-PAGE gels were used to clearly separate the LC3-I and LC3-II bands. For GFP-LC3 puncta detection, H1581 and H520 cells were transduced by a lentiviral vector to stably express GFP-LC3. Numbers of GFP-LC3 puncta per cell in cells transfected with GFP-LC3 were quantified by fluorescence microscopy. All GFP-LC3 puncta quantitation was performed by an observer blinded to experimental conditions.

### MDC staining

Cells were seeded in 24-well plates with sterile cover slips. After incubation with 50 μM MDC in growth medium for 30 min at 37 °C, cells were washed three times with PBS, and fixed in 4% PFA for 30 min at RT. MDC was observed with a 335 (380)/525 nm filter set by fluorescence microscopy (Leica, Germany).

### Lentiviral experiments

The GFP-MAP1LC3B plasmid was purchased from Obio Technology Corp., Ltd. GFP-LC3 lentiviruses were produced from pLOV-PuroR-based vectors by co-transfected with delta and VSVG in 293 T cells. After transduction, H1581/GFP-LC3 and H520/GFP-LC3 cell lines were generated by selection with 1 μg/ml puromycin.

Lentiviral short hairpin RNA (shRNA) clones targeting FGFR1 and non-targeting control were purchased from Hanbio. H1581 and H520 cells were transduced by lentiviral-encoded shRNA targeting RFP as a control or two independent shRNAs against FGFR1 respectively, and then selected for puromycin resistance. All sequences are listed in Additional file 1: Table S1.

### Small interfering RNA and transfection

Two 21-mer oligonucleotide small interfering RNA (siRNA) duplexes targeting FGFR1 and a negative control siRNA were acquired from GenePharma. Two 21-mer oligonucleotide siRNA duplexes targeting beclin-1 and a negative control siRNA were acquired from Biotend. H1581 and H520 cells were transfected with siRNA using Lipofectamine RNAiMAX transfection reagent (Invitrogen) according to the manufacturer’s instructions. The sequences targeting FGFR1 and beclin-1 are given in Additional file 2: Table S2.

### DNA constructs and transfection

Constitutively active Akt1 was a gift from Heng Zhao [29]. Constitutively active MEK (S218D/S222D), and FGFR1 construct were ordered from Addgene [30]. Plasmid transfection was performed using jetPRIME (Polyplus) according to the manufacturer’s instructions.

### Cell viability assay

Cell viability was measured using Cell Counting Kit-8 (CCK-8, Dojindo Laboratories, Japan). Cells were seeded in 96-well plates at the density of 1 × 10^4^/well, and then exposed to beclin-1 siRNA or AZD4547 at indicated concentrations. CCK-8 solution was added to the medium at a dilution of 1:10 and cells were incubated with CCK-8 solution for 1–2 h at 37 °C. The absorbance at 450 nm was measured using a microplate reader (Synergy2, BioTek).

### Apoptosis assay

Apoptosis was measured using FITC Annexin V apoptosis detection kit I (BD Biosciences) according to the manufacturer’s instructions. Cells were harvested, centrifuged, and then resuspended in 100 μl of 1 X binding buffer containing 5 μl of FITC Annexin V and 5 μl propidium iodide (PI) for 15 min at RT in the dark. After incubation with 400 μl 1 X binding buffer, the cells were analyzed by flow cytometry (BD FACSCanto II) and data were evaluated using FlowJo software.

### Analysis of publicly available database

To examine the correlation between beclin-1 expression and MEK1 phosphorylation in lung cancer patients, we downloaded data from TCGA (http://www.cbioportal.org/public-portal/), and analyzed data using GraphPad Prism software [31, 32].

To analyze the prognostic value of LC3B mRNA expression on lung cancer patients, we generated Kaplan-Meier survival curves based on expression values [33].

### Statistical Analysis

Results are expressed as mean ± standard deviation (SD) or mean ± standard error of mean (SEM) collected from at least three independent experiments. Data were analyzed by GraphPad Prism software with one-way ANOVA or Student *t* test. The relationship between beclin-1 expression and MEK1 phosphorylation was examined by Pearson correlation. A value of *p* < 0.05 was considered statistically significant.

## Results

### FGFR1 activation inhibits autophagy

**Fig. 1:**
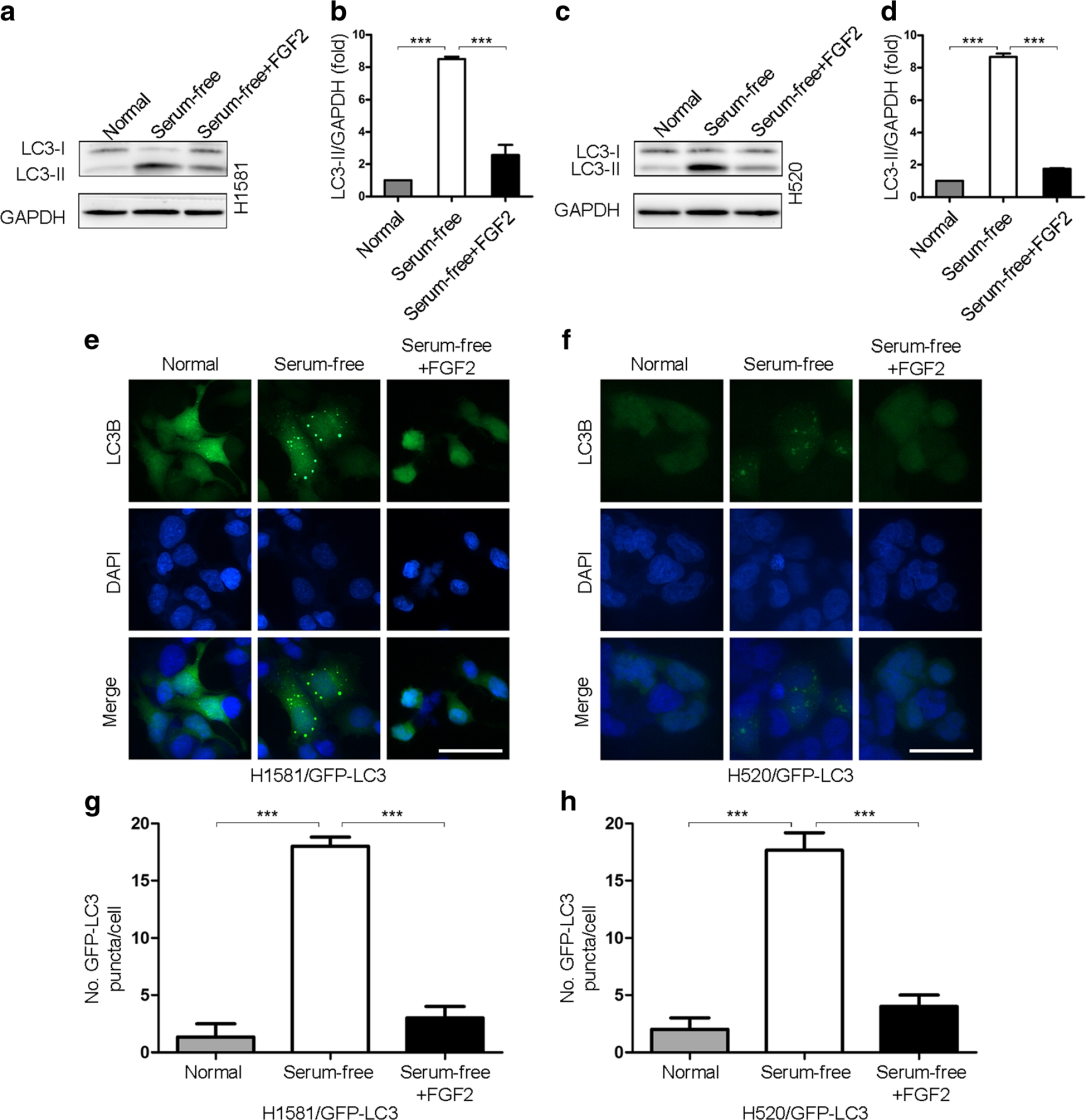
FGFR1 activation inhibits autophagy. **a** LC3-I/II western blot analysis in H1581 NSCLC cells cultured O/N in normal medium, serum-free medium, or serum-free medium plus FGF2 (25 ng/ml, 2 h). **b** Quantification of LC3-II levels in (**a**); mean ± SD, *n =* 3, ***p < 0.001. **c** LC3-I/II western blot analysis in H520 cells in conditions shown in (**a**). **d** Quantification of LC3-II levels in (**c**); mean ± SD, *n =* 3, ***p < 0.001. **e** Representative images of GFP-LC3 puncta in H1581/GFP-LC3 cells in conditions shown in (**a**). Scale bars represent 25 μm. **f** Representative images of GFP-LC3 puncta in H520/GFP-LC3 cells in conditions shown in (**c**). Scale bars represent 25 μm. **g** Quantitation of GFP-LC3 puncta in conditions shown in (**e**); mean ± SD, *n =* 3, ***p < 0.001. **h** Quantitation of GFP-LC3 puncta in conditions shown in (**f**); mean ± SD, *n =* 3, ***p < 0.001

To evaluate whether FGFR1 activation inhibits autophagy, we used two human NSCLC cell lines, H1581 and H520, both of them are FGFR1 gene amplification and high FGFR1 protein expression (Additional file 3: Figure S1) [34]. We detected autophagy by measuring levels of LC3-II, the autophagosome-associated lipidated form of LC3, and by examining the subcellular localization of a green fluorescent autophagy reporter protein, GFP-LC3 [26]. Serum depletion resulted in marked autophagy induction, as evidenced by increased LC3-II conversion (Fig. 1a-d) and an increase in GFP-LC3 puncta per cell (Fig. 1e-h), which was reversed by FGF2 addition to the serum-starved cells for 2 h.

We further employed MDC staining to verify inhibition of autophagy by FGF2. As shown in Additional file 4: Figure S2, serum depletion induced the accumulation of MDC in the cytoplasmic vacuoles in H1581 and H520 cells, which was reversed by FGF2 addition to the serum-starved cells for 2 h.

### FGFR1 inhibition induces autophagic activity

**Fig. 2:**
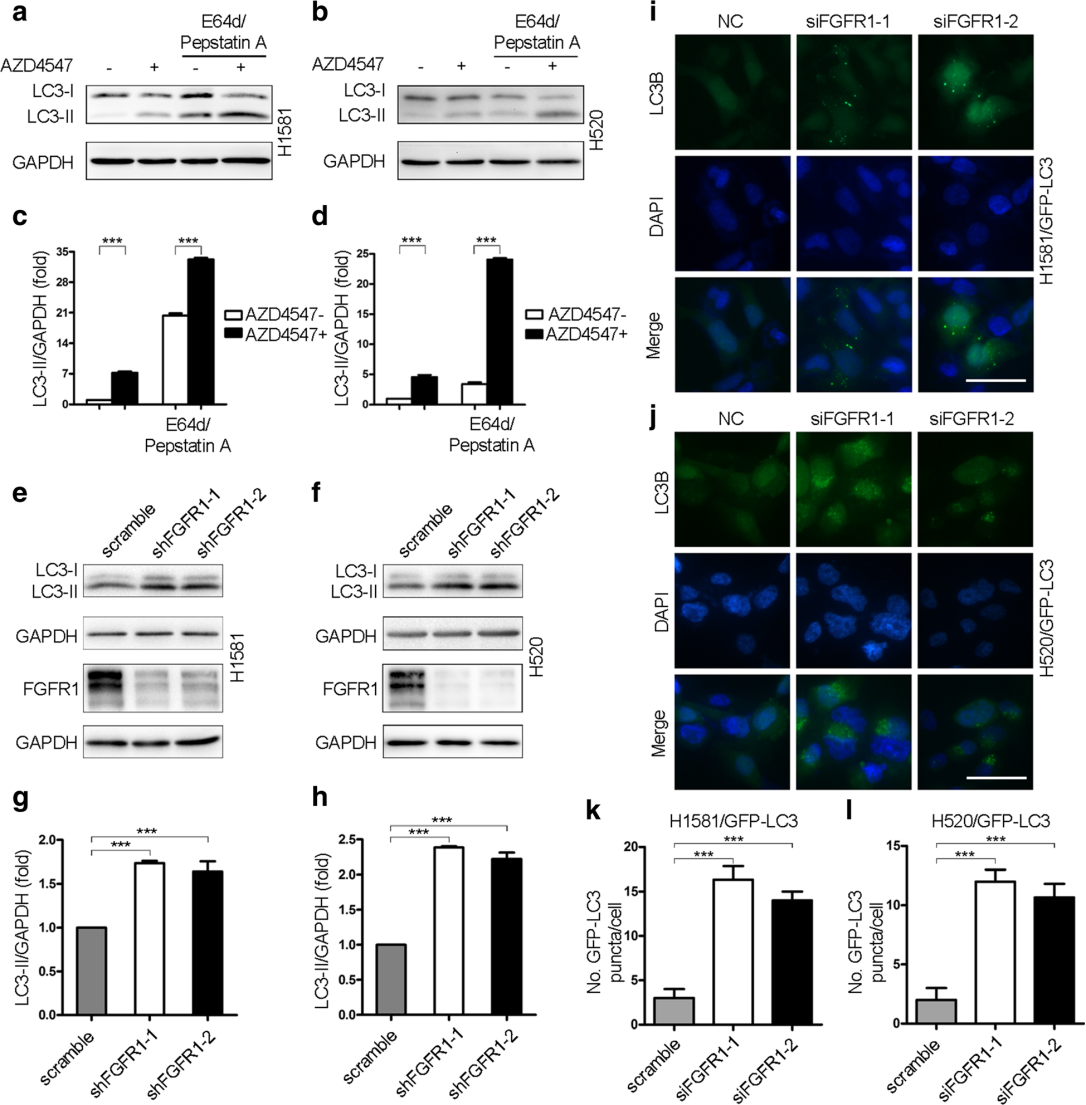
FGFR1 inhibition induces autophagic activity. **a** H1581 cells were treated with 1 μM AZD4547 for 24 h. The last 8 h was in the presence or absence of E64d (10 μg/ml) and pepstatin A (10 μg/ml). Protein levels of LC3-I/II, and GAPDH were measured by western blot assays. **b** H520 cells were treated with 1 μM AZD4547 for 24 h. E64d and pepstatin A were added to the cells 8 h before harvesting. **c** Quantification of LC3-II levels in (**a**); mean ± SEM, *n =* 3, ***p < 0.001. **d** Quantification of LC3-II levels in (**b**); mean ± SEM, *n =* 3, ***p < 0.001. **e** The levels of LC3-II and GAPDH were determined by western blot in H1581 cells stably transduced by shRNA targeting FGFR1 and a control shRNA. FGFR1 and GAPDH protein expression levels by western blot were included as a control for the efficiency of shRNA knockdown. **f** Confirmation of changes in levels of autophagy after knockdown of FGFR1 by western blot with antibodies against LC3-I/II in H520 cells. **g** Quantification of LC3-II levels in (**e**); mean ± SD, *n =* 3, ***p < 0.001. **h** Quantification of LC3-II levels in (**f**); mean ± SD, *n =* 3, ***p < 0.001. **i** H1581/GFP-LC3 cells transfected with either siRNA targeting FGFR1 or negative control (NC) were analyzed by fluorescence microscopy to determine the distribution of punctate GFP-LC3. E64d and pepstatin A were added for 8 h before fixation. Scale bars represent 25 μm. **j** Punctate GFP-LC3 distribution of H520/GFP-LC3 cells transfected with two independent FGFR1 siRNA constructs or a negative control. E64d and pepstatin A were added for 8 h before fixation. Scale bars represent 25 μm. **k** Quantification of levels of autophagy in H1581/GFP-LC3 cells transfected with control siRNA or siRNA against FGFR1 in conditions shown in (**i**); mean ± SD, *n =* 3, ***p < 0.001. **l** Quantification of data from (**j**); mean ± SD, *n =* 3, ***p < 0.001

To examine the effect of FGFR1 inhibition on autophagy in FGFR1-amplified NSCLC cell lines, we treated H1581 and H520 cells with 1 μM AZD4547. As shown in Fig. 2a-d, AZD4547 resulted in a minor increase in the level of LC3-II. However, an increased level of LC3-II is not a measure of autophagic flux per se, but can reflect both the induction of autophagy and/or inhibition of autophagosome clearance [26]. To further investigate the magnitude of the flux through the autophagic pathway, cells were treated in the presence of the lysosomal protease inhibitors E64d and pepstatin A. Thus AZD4547 induced a more pronounced accumulation of LC3-II in the presence of lysosomal protease inhibitors (Fig. 2a and b, right panel). This indicated enhanced autophagic flux and not simply blocked autophagic degradation. Lysosomal protease inhibitors were included in all subsequent experiments.

To confirm the effect of FGFR1 inhibition on autophagy in FGFR1-amplified NSCLC cell lines, we transduced H1581 and H520 cells by lentiviral vectors encoding a control shRNA targeting RFP or two independent shRNAs targeting FGFR1. Using two well established markers of this cellular process, we demonstrated that knockdown of FGFR1 induced autophagy by the increase in the level of LC3-II in H1581 and H520 cells (Fig. 2e-h). FGFR1 silencing increased GFP-LC3 puncta in H1581 cells stably transfected with GFP-LC3 (Fig. 2i and k), and in H520/GFP-LC3 cells (Fig. 2j and l). In addition to utilization of LC3-II accumulation and GFP-LC3 puncta formation as markers of autophagy, we further explored MDC staining to confirm induction of autophagy by AZD4547. As shown in Additional file 5: Figure S3, treatment with AZD4547 induced the accumulation of MDC in the cytoplasmic vacuoles in both cell lines. Conversely, overexpression of FGFR1 in cultured SK-MES-1 NSCLC cells (with low FGFR1 expression) led to a decrease in LC3-II (Additional file 6: Figure S4). All these suggest that pharmacological or genetic inhibition of FGFR1 induces autophagic activity in FGFR1-amplified NSCLC cells.

### FGF2 activates the ERK/MAPK pathway

Since the MEK/ERK and PI3K/AKT/mTOR are two major pathways downstream of FGFR1, we explored whether the main downstream signaling axis was activated by FGF2. ERK1/2 phosphorylation was induced within 30 min after treatment with FGF2 at a final concentration of 25 ng/ml. The phosphorylation was reduced at a lagging mode in H1581 cells (Additional file 7: Figure S5A). Consistently, ERK1/2 was also phosphorylated within 30 min, which was then gradually reduced in H520 cells (Additional file 7: Figure S5B). However, there were no obvious changes in the phosphorylation of AKT and Stat3 (data not shown). These results suggest that FGF2 induces activation of the ERK/MAPK pathway.

### The induction of autophagy by FGFR1 inhibition is mediated through suppressing the ERK/MAPK pathway

**Fig. 3:**
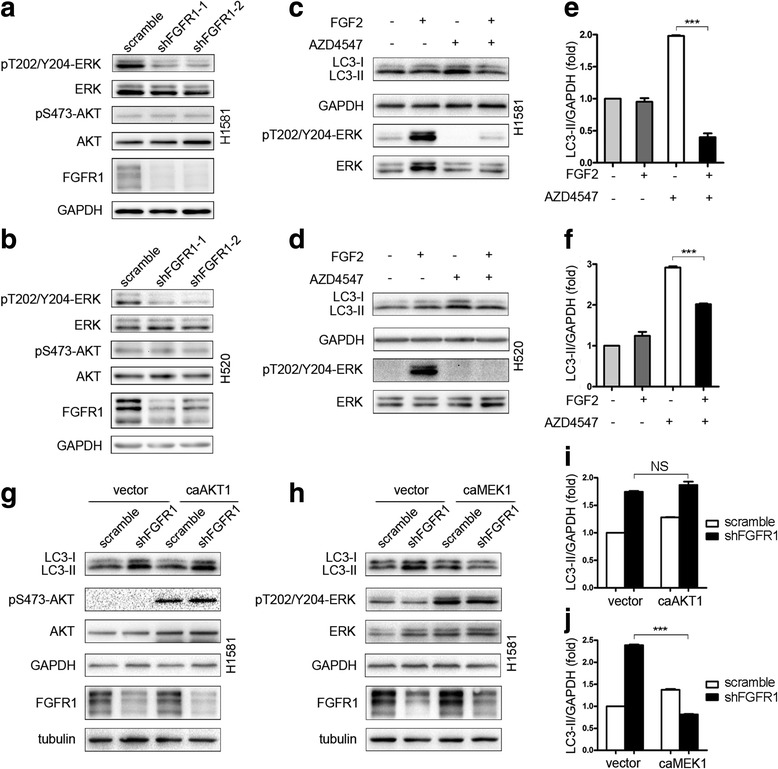
Constitutive activation of ERK/MAPK pathway, but not PI3K pathway, prevents autophagy induced by FGFR1 inhibition. **a** and **b** ERK and AKT phosphorylation by western blot in H1581 and H520 cells stably transduced by two independent FGFR1 shRNA constructs or a non-targeting control vector. FGFR1 protein expression levels were included as a control of knockdown efficiency. The filters were stripped and reprobed for total ERK and AKT to ensure equal loading of cell protein in each lane. **c** H1581 cells were treated with 1 μM AZD4547 for 24 h. FGF2 (25 ng/ml) was added for 2 h before harvesting. **d** H520 cells were treated with AZD4547 (1 μM) for 24 h. Cells were treated with FGF2 for 2 h before harvesting and then subjected to western blotting analysis. **e** Quantification of LC3-II levels in (**c**); mean ± SD, *n =* 3, ***p < 0.001. **f** Quantification of LC3-II levels in (**d**); mean ± SD, *n* = 3, ***p < 0.001. **g** Western blot showing that the expression of a caAKT1 construct failed to prevent increase in LC3-II induced by shFGFR1 in H1581 cells. **h** Western blot showing that the expression of a caMEK1 prevented increase in LC3-II induced by shFGFR1 in H1581 cells. **i** Quantification of LC3-II levels in (**g**); mean ± SEM, *n* = 3, NS, not significant. **j** Quantification of LC3-II levels in (**h**); mean ± SEM, *n* = 3, ***p < 0.001

Downregulation of FGFR1 by transduction of FGFR1-specific shRNAs inhibited ERK1/2 phosphorylation, but no effect on phosphorylation of AKT in H1581 and H520 cells (Fig. 3a and b). In addition, ERK phosphorylation activated by FGF2 prevented AZD4547-induced increase in LC3-II in H1581 and H520 cells (Fig. 3c-f). These data partially indicated that inhibition of the ERK/MAPK signaling pathway was required for the induction of autophagy after AZD4547 treatment.

To provide further evidence, we found that the increase of LC3-II induced by shFGFR1 in H1581 cells was not affected after transient transfection with a constitutively active AKT1 (caAKT1) construct (Fig. 3g and i). In contrast, the increase of LC3-II induced by shFGFR1 in H1581 cells was prevented by transient transfection of H1581 cells with a constitutively active MEK1 (caMEK1) construct (Fig. 3h and j). These results indicate that the induction of autophagy after FGFR1 inhibition is mediated through suppressing the ERK/MAPK pathway, but not the PI3K/AKT pathway.

### FGFR1 inhibition induces autophagy through beclin-1

**Fig. 4:**
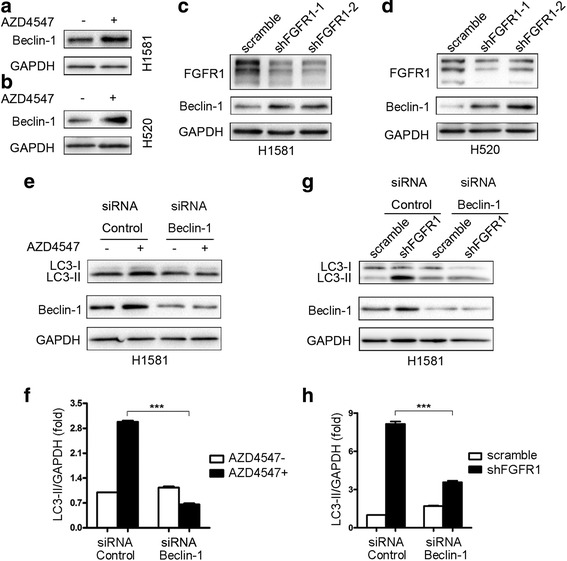
Downregulation of beclin-1 prevents autophagy induced by FGFR1 inhibition. **a** and **b** H1581 and H520 cells were treated with 1 μM AZD4547 for 24 h. Then, total lysates were harvested and subjected to western blot analysis. **c** and **d** H1581 and H520 cells, stably transduced by the indicated lentiviruses (scramble or shFGFR1), were subjected to western blotting analysis. **e** H1581 cells were transfected with beclin-1 siRNA or control siRNA using Lipofectamine RNAiMAX transfection reagent as described in materials and methods. At 24 h after transfection, the cells were treated with or without AZD4547 (1 μM) for 24 h. **f** Quantification of LC3-II levels in (**e**); mean ± SEM, *n* = 3, ***p < 0.001. **g** H1581 cells, stably transduced by the indicated lentiviruses (scramble or shFGFR1), were transfected with beclin-1 siRNA or control siRNA, and then cells were subjected to western blotting analysis. **h** Quantification of LC3-II levels in (**g**); mean ± SEM, *n* = 3, ***p < 0.001

As shown in Fig. 4a and b, AZD4547 treatment upregulated the expression of beclin-1 in H1581 and H520 cells. Similarly, knockdown of FGFR1 increased the level of beclin-1 in stably transduced H1581 and H520 cells (Fig. 4c and d). To gain further insight into the mechanism, we examined the role of beclin-1 in FGFR1 inhibition-induced autophagy. AZD4547-induced autophagy was inhibited by beclin-1 silencing in H1581 cells (Fig. 4e and f). Furthermore, inhibition of beclin-1 prevented LC3-II accumulation induced by shFGFR1 (Fig. 4g and h). These results indicate that FGFR1 inhibition induced autophagy through beclin-1 in FGFR1-amplified NSCLC cells.

### Upregulation of beclin-1 by FGFR1 inhibition contributes to the induction of autophagy through inhibiting ERK pathway

**Fig. 5:**
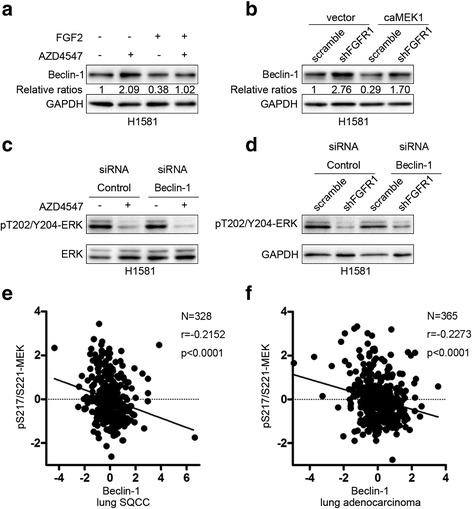
Upregulation of beclin-1 by FGFR1 inhibition contributes to induction of autophagy through inhibiting ERK pathway. **a** H1581 cells were treated with AZD4547 (1 μM) for 24 h prior to treatment with or without FGF2 (25 ng/ml) for 2 h. After treatment, cells were harvested and subjected to western blotting analysis. **b** H1581 cells, stably transduced by FGFR1 shRNA or control shRNA, were transfected with caMEK1 plasmid or control vector using jetPRIME transfection reagent as described in materials and methods. At 24–48 h after transfection, the cells were harvested and then subjected to western blotting analysis. **c** H1581 cells were transfected with beclin-1 siRNA or control siRNA using Lipofectamine RNAiMAX transfection reagent. At 24 h after transfection, the cells were treated with or without AZD4547 (1 μM) for 24 h. **d** H1581 cells, stably transduced by the indicated lentiviruses (scramble or shFGFR1), were transfected with beclin-1 siRNA or control siRNA, and then cells were subjected to western blotting analysis. **e** MEK phosphorylation was analyzed in a cohort of lung SQCC patients that had altered levels of beclin-1 protein expression using cBioPortal. **f** MEK phosphorylation was analyzed in a cohort of lung adenocarcinoma patients that had altered levels of beclin-1 expression using TCGA database

Activation of ERK activity by FGF2 (Fig. 5a) or transient transfection with a caMEK1 construct (Fig. 5b) blocked upregulation of beclin-1 by FGFR1 inhibition. Activation of ERK/MAPK pathway prevented LC3-II accumulation induced by FGFR1 inhibition (Fig. 3c and h). These indicate that pharmacological or genetic inhibition of FGFR1 increased level of beclin-1, which is mediated at least in part through inhibiting ERK/MAPK pathway.

Downregulation of beclin-1 suppressed the induction of autophagy by FGFR1 inhibition (Fig. 4e and g) but did not affect inhibition of ERK phosphorylation by AZD4547 or shFGFR1 (Fig. 5c and d), indicating that beclin-1 functions downstream of the ERK/MAPK pathway.

To assess the impact of beclin-1 on MEK1 activation, we analyzed the correlation between beclin-1 expression and MEK1 phosphorylation in a cohort of lung cancer patients that had altered levels of beclin-1 protein expression as measured by reverse-phase protein array (RPPA) [31, 32]. A statistically negative correlation between beclin-1 and p-MEK1 (S217/S221, Pearson’s r = −0.2152) was observed in a cohort of lung SQCC samples (Fig. 5e). Inverse correlation of beclin-1 expression and MEK1 phosphorylation (S217/S221, Pearson’s r = −0.2273) was also observed in lung adenocarcinoma patients (Fig. 5f), which supported our preclinical data.

### Inhibition of autophagy by beclin-1 silencing enhances apoptosis after AZD4547 treatment

**Fig. 6:**
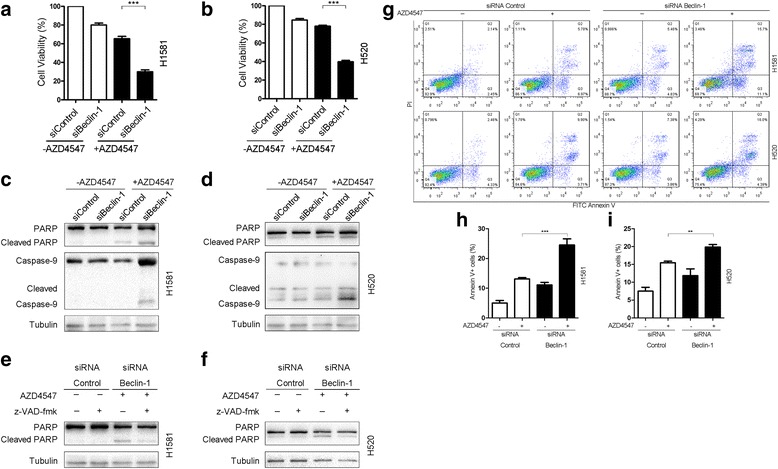
Inhibition of autophagy by beclin-1 silencing enhances apoptosis after AZD4547 treatment. **a** H1581 cells were transfected with beclin-1 siRNA or control siRNA using Lipofectamine RNAiMAX transfection reagent. At 24 h after transfection, the cells were treated with or without AZD4547 (1 μM) for 24 h. Cell viability was determined by CCK-8 assay; mean ± SD, *n* = 3, ***p < 0.001. **b** H520 cells were treated as described in (**a**), and cell viability was measured by CCK-8 assay; mean ± SD, *n* = 3, ***p < 0.001. **c** and **d** H1581 and H520 cells transfected with beclin-1 siRNA or control siRNA were treated with or without AZD4547 (1 μM) for another 24 h. Cleavage of both PARP and caspase-9 were analyzed by western blot assay. **e** and **f** H1581 and H520 cells transfected with beclin-1 siRNA or control siRNA were treated with or without AZD4547 (1 μM) for another 24 h. The last 5 h was in the presence or absence of z-VAD-fmk (20 μM). PARP cleavage and tubulin were analyzed by western blot assay. **g** H1581 and H520 cells transfected with beclin-1 siRNA or control siRNA were treated with or without AZD4547 (1 μM) for another 24 h. Cell apoptosis was determined by flow cytometry using FITC Annexin V/PI staining. The horizontal and vertical axes represent labeling with FITC Annexin V/PI, respectively. LR (Q3) represents early apoptotic cells (positive for Annexin V only), UR (Q2) represents late apoptotic cells (positive for both Annexin V and PI), and LL (Q4) represents live cells. **h** H1581 cells were treated as described in (**g** *upper panel*). The number of early/late apoptotic cells is shown as the sum of Annexin V positive and Annexin V/PI double positive cells; mean ± SD, *n* = 3, ***p < 0.001. **i** H520 cells were treated as described in (**g** *lower panel*); mean ± SD, *n* = 3, **p < 0.01

To establish whether autophagy inhibition would be an effective therapeutic intervention after AZD4547 treatment, we examined the effect of beclin-1 silencing on AZD4547-induced cell death. In the absence of AZD4547, treatment of cells with beclin-1 siRNA led to a decrease in cell viability (Fig. 6a and b, left panel). In the presence of AZD4547, substantial decrease in cell viability was obtained (Fig. 6a and b, right panel). These indicate that inhibition of autophagy enhances cell death after AZD4547 treatment.

To determine the role of autophagy in cell growth and survival after AZD4547 treatment, we examined the effect of autophagy inhibition on apoptosis. Western blot assays showed that the combination of AZD4547 with beclin-1 silencing noticeably increased the cleavage of poly (ADP-ribose) polymerase (PARP) and caspase-9 in H1581 and H520 cells (Fig. 6c and d), suggesting that this combination activated the apoptosis pathway. As shown in Fig. 6e and f, the pan-caspase inhibitor, z-VAD-fmk blocked the cleavage of PARP in both cells. Flow cytometry analysis showed that the proportions of early apoptotic cells (FITC Annexin V positive and PI negative) and late apoptotic cells (FITC Annexin V positive and PI positive) increased when NSCLC cells were cotreated with AZD4547 and beclin-1 siRNA (Fig. 6g-i). These data indicated that the type of cell death induced by combination of AZD4547 and beclin-1 silencing was partially caspase-dependent apoptosis.

### High levels of LC3B mRNA in lung cancer are associated with poor prognosis

**Fig. 7:**
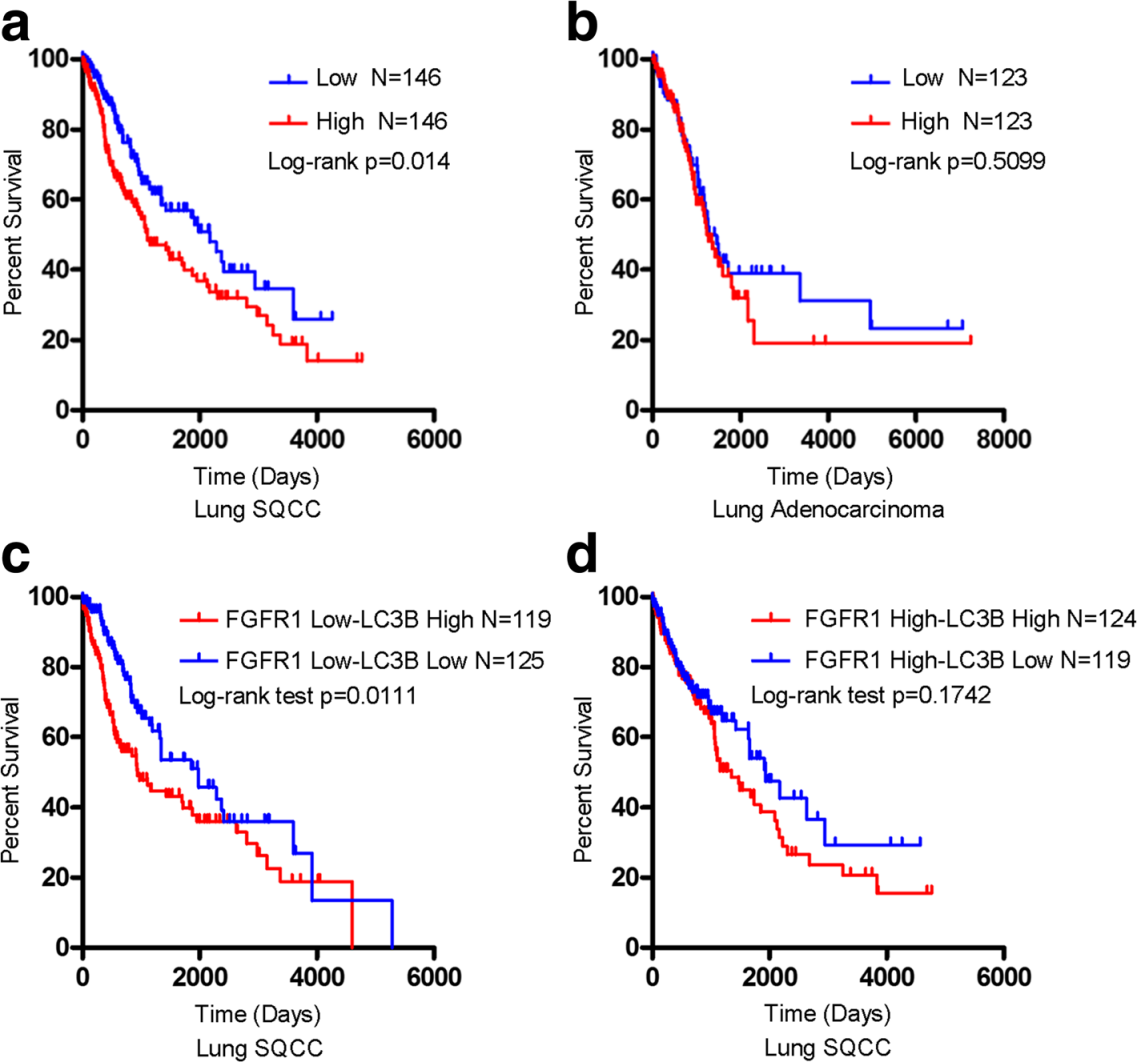
High levels of LC3B mRNA are associated with poor prognosis in NSCLC patients. **a** and **b** Kaplan-Meier *curves* of OS, with respect to LC3B mRNA level, in **a** lung SQCC (*N* = 146 for low expression and *N* = 146 for high expression, *p* = 0.014), and **b** lung adenocarcinoma (*N* = 123 for low expression and *N* = 123 for high expression, *p* = 0.5099). **c** Kaplan-Meier curves for OS in FGFR1 low - LC3B low expression (*N* = 125) and FGFR1 low - LC3B high expression (*N* = 119) patients, the latter had poorer OS (*p* = 0.0111). **d** Kaplan-Meier curves for OS in FGFR1 high - LC3B low expression (*N* = 119) and FGFR1 high - LC3B high expression (*N* = 124) patients, the latter conferred decreased OS (*p* = 0.1742). P-values are based on the log-rank test (**a-d**)

To explore the role of autophagy among distinct subtypes of lung cancer, we analyzed the expression of LC3B and performed analyses based on log-rank test. High LC3B expression was a marker of poor prognosis in both lung SQCC (Fig. 7a) and adenocarcinoma (Fig. 7b) (expression data are described in http://www.oncolnc.org/) [33]. We also confirmed the prognostic value of LC3B in lung cancer patients from The Cancer Genome Atlas (TCGA) cohort (expression data are described in http://www.cbioportal.org/public-portal/) [31, 32]. Lung cancer patients with alterations in LC3B exhibited lower overall survival (OS) than those without alterations in LC3B (Additional file 8: Figure S6).

To further explore the prognostic value of LC3B in lung SQCC patients, we stratified them for high vs. low expression of FGFR1 and LC3B, respectively [33]. In low FGFR1-expressing lung SQCC, high LC3B expression had significantly poorer OS compared with low LC3B expression (Fig. 7c). In high FGFR1-expressing lung SQCC, high LC3B expression conferred worse OS in comparison to low LC3B expression (Fig. 7d).

**Fig. 8:**
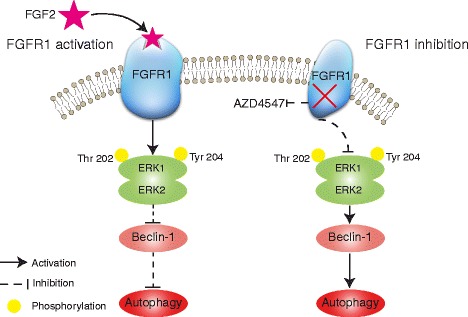
A schema depicting a mechanism by which FGF2/FGFR1 regulates autophagy. *Left panel*: FGFR1 activation by FGF2 upregulates ERK1/2 phosphorylation and then downregulates beclin-1, thereby suppresses autophagy. *Right panel*: FGFR1 inhibition (AZD4547 or FGFR1 knockdown) downregulates phosphorylation of ERK1/2 and subsequently upregulates beclin-1, thereby induces autophagy

Based on the study presented herein, we propose a novel mechanism by which FGF2/FGFR1 regulates autophagy in FGFR1-amplified NSCLC cells (Fig. 8).

## Discussion

FGFR1 is frequently amplified in lung SQCC and is a therapeutic target under investigation in multiple solid tumors [35]. Clinical application of FGF/FGFR-targeted therapy is under development for the treatment of cancers caused by aberrant FGF signaling. FGFR inhibitors mainly target the cytoplasmic kinase domain, whereas a few FGF inhibitors target the extracellular ligand-binding region [36]. Patients with FGFR genetic alterations are predicted to be appropriate candidates for FGFR inhibitors-based therapy. Treatment with a single tyrosine kinase inhibitor (TKI) represents a step toward personalized cancer therapy, but intrinsic and acquired resistance limit their long-term benefit. What determines response to FGFR inhibition in FGFR-amplified cancers is unknown.

It is proposed that there are at least four functional forms of autophagy, cytoprotective, cytotoxic, cytostatic, and nonprotective [37]. The role of autophagy in cancer is paradoxical as it functions both as a tumor suppressor and as a drug resistance mechanism [14]. On one hand, autophagy appears to function as a tumor suppressor mechanism as defective autophagy is associated with malignant transformation and carcinogenesis. Studies have demonstrated that heterozygous disruption of beclin-1 promotes tumorigenesis while the overexpression inhibits tumorigenesis [12, 13]. In this circumstance, the induction of autophagy may help to reverse the malignant phenotype. On the other hand, conventional chemotherapeutic drugs, radiation and the hypoxic tumor environment can promote a cytoprotective form of autophagy in tumor cells [38]. Consequently interference with or suppression of this autophagy will be used as a therapeutic approach. Autophagy and apoptosis are tightly regulated biological processes and their cross-talk is complex, with conflicting models of interplays being indicated [39–41]. Our study indicated that suppression of autophagy promoted apoptosis after AZD4547 treatment.

This study is designed to test the hypothesis that FGFR inhibitor AZD4547 induced autophagy in FGFR1-amplified NSCLC cells. Herein we found that genetic inhibition of autophagy (beclin-1 silencing) enhanced apoptosis after AZD4547 treatment in H1581 and H520 cells. AZD4547 induced protective autophagy in FGFR1-amplified NSCLC cells. Based on the above findings, we analyzed human lung cancer database and confirmed that lung SQCC with high LC3B levels conferred poor prognosis [31–33].

There are multiple links between oncogene and autophagy. Firstly, activated EGFR directly phosphorylates and inhibits beclin-1, a key component in autophagy initiation [42]. Secondly, EGFR TKIs upregulate autophagy in many cancer cells [43]. These studies support a role for EGFR signaling in autophagy suppression. A high-throughput image-based screening and analysis approach indicate that knockdown of FGFR1 increases autophagy flux [44]. FGFR1 TKI induces protective autophagy through suppressing AKT/mTOR signaling pathway in FGFR1-amplified breast cancer cell lines [45]. Similarly, our studies demonstrate that FGFR1 inhibition promotes induction of autophagy.

Based on earlier studies that TKIs induce autophagy [46] and the belief that autophagy induction may lead to chemoresistance [47], there are currently several NIH-sponsored clinical trials that combine autophagy inhibitory agents with TKIs in the treatment of NSCLC. Previous studies have claimed that autophagy induction can limit or enhance the response to TKI therapy. In our study, AZD4547 treatment induced autophagic activity in both of the cells, suggesting that synergy with autophagy inhibition is a sequence of the drug itself inducing autophagy. Combining autophagy inhibitors with FGFR1 selective inhibitors identifies a potential strategy for treatment of FGFR1-amplified cancers. This is in contrast to a recent study, which has shown that autophagy induction contributes to EGFR TKI responses in NSCLC with active EGFR mutations, and the use of autophagy inhibitors in patients receiving EGFR TKI may adversely (rather than favorably) affect their clinical course [42].

Activation of ERK has been associated with either induction or inhibition of autophagy, depending on cell types [48–50]. Here we demonstrate that inhibition of the ERK/MAPK pathway was required for FGFR1 inhibition-induced autophagy, because constitutive activation of the ERK/MAPK pathway by expression of a caMEK1 construct prevented the induction of autophagy by FGFR1 inhibition.

A few caveats should be clarified. Our findings are based on in vitro experiments and lack in vivo studies that directly assess the effects of modulating autophagy on the response of NSCLC to TKIs. For the future, it will be necessary to determine whether FGFR1 inhibition-induced autophagy can be targeted for therapeutic gains using in vivo tumor models, particularly patient derived xenograft (PDX) models. The beclin-1/VPS34 complex plays a crucial role in autophagosome formation [51]. Using TCGA database, we observed an inverse correlation between beclin-1 expression and MEK phosphorylation in lung cancer samples, which supported our preclinical data. However, we do not explore the regulation of the beclin-1 interactome such as bcl-2/beclin-1 or beclin-1/VPS34 complex. It will be important to explore the mechanisms in further study.

In a summary, we found that pharmacological or genetic inhibition of FGFR1 induced autophagy; the response was elicited through complex mechanisms involving both inhibition of the ERK/MAPK pathway and upregulation of beclin-1. Furthermore, our findings indicate that clinical efficacy of treatments for FGFR1-driven lung cancer may be achieved by combining autophagy inhibitors and FGFR-specific TKIs. Further in-depth studies are needed to understand the role of autophagy in FGFR1-targeted therapy, which may benefit patients through development of novel combination treatments and provide the basis for the design of multiple clinical trials.

## Conclusions

In this study, we offer convincing evidence that autophagy is induced in FGFR1-amplified NSCLC cells after pharmacological or genetic inhibition of FGFR1. This effect is dependent on beclin-1 through suppressing ERK/MAPK pathway. These indicate a new mechanism by which FGF2/FGFR1 regulates autophagy. Synergistic targeting of autophagy and FGFR pathways should be further explored in FGFR1-amplified NSCLC in vivo.

## Additional files

Additional file 1: Table S1. Sequences of FGFR1 shRNA constructs that were used in the study. (TIF 65 kb)

Additional file 2: Table S2. Sequences of all siRNA constructs that were used in the study. (TIF 67 kb)

Additional file 3: Figure S1. Immunoblot analysis of FGFR1 in NSCLC cell lines. Extracts from NSCLC cells were subjected to immunoblot analysis for FGFR1, GAPDH as a loading control. (TIF 140 kb)

Additional file 4: Figure S2. FGFR1 activation inhibits autophagy. (A) MDC staining analysis in H1581 (upper panel) and H520 (lower panel) cells cultured O/N in normal medium, serum-free medium, or serum-free medium plus FGF2 (25 ng/ml, 2 h). Scale bars represent 25 μm. (B) Quantitative results of MDC staining in conditions shown in (A); mean ± SD, *n* = 3, ***p < 0.001, **p < 0.01. (TIF 2400 kb)

Additional file 5: Figure S3. AZD4547 induces autophagy. (A) H1581 and H520 cells grown in 24-well plates were treated with DMSO, or AZD4547 (1 μM) for 24 h. MDC staining was performed and the cells were examined under a fluorescence microscope. Scale bars represent 25 μm. (B) Quantitative results of MDC staining in conditions shown in (A); mean ± SD, *n* = 3, **p < 0.01. (TIF 1577 kb)

Additional file 6: Figure S4. Overexpression of FGFR1 inhibits autophagy in SK-MES-1 cells. (A) The SK-MES-1 cells were transfected with control vector or FGFR1 plasmid. At 48 h post-transfection, LC3-I/II and GAPDH were determined by western blot. The lower panel shows the efficiency of FGFR1 overexpression. (B) Quantification of LC3-II levels in (A); mean ± SD, *n* = 3, ***p < 0.001. (TIF 321 kb)

Additional file 7: Figure S5. FGF2 activates the ERK/MAPK pathway. (A and B) H1581 and H520 cells treated with 25 ng/ml FGF2 were lysed and probed with the indicated antibodies. The filters were stripped and reprobed for total FGFR1 and ERK to ensure equal loading of cell protein in each lane. (TIF 899 kb)

Additional file 8: Figure S6. Kaplan-Meier curves for lung cancer patients. OS for patients in TCGA cohort, in lung cancer (*N* = 12 for cases with alterations in LC3B and *N* = 941 for cases without alterations in LC3B). P-values are based on the log-rank test. (TIF 179 kb)

## Abbreviations

caMEK1: Constitutively active MEK1
DMSO: Dimethyl sulfoxide
ERK: Extracellular signal-activated kinase
FBS: Fetal bovine serum
FGF2: Fibroblast growth factor 2
FGFR1: Fibroblast growth factor receptor 1
FRS2α: FGF receptor substrate 2α
LC3: Microtubule-associated protein 1 light chain 3
MDC: Monodansylcadaverine
NSCLC: Non-small cell lung cancer
PARP: Poly (ADP-ribose) polymerase.
PBS: Phosphate buffered saline
PFA: Paraformaldehyde
RPPA: Reverse-phase protein array
RTK: Receptor tyrosine kinase
shRNA: Short hairpin RNA
siRNA: Small interfering RNA
SQCC: Squamous cell carcinoma

## References

[1] Siegel RL, Miller KD, Jemal A (2016). Cancer Statistics, 2016. Ca-a Cancer Journal for Clinicians.

[2] Drilon A, Rekhtman N, Ladanyi M, Paik P (2012). Squamous-cell carcinomas of the lung: emerging biology, controversies, and the promise of targeted therapy. Lancet Oncol.

[3] Dieci MV, Arnedos M, Andre F, Soria JC (2013). Fibroblast growth factor receptor inhibitors as a cancer treatment: from a biologic rationale to medical perspectives. Cancer Discov.

[4] Carter EP, Fearon AE, Grose RP (2015). Careless talk costs lives: fibroblast growth factor receptor signalling and the consequences of pathway malfunction. Trends Cell Biol.

[5] Weiss J, Sos ML, Seidel D, Peifer M, Zander T, Heuckmann JM, Ullrich RT, Menon R, Maier S, Soltermann A (2010). Frequent and focal FGFR1 amplification associates with therapeutically tractable FGFR1 dependency in squamous cell lung cancer. Sci Transl Med.

[6] Dutt A, Ramos AH, Hammerman PS, Mermel C, Cho J, Sharifnia T, Chande A, Tanaka KE, Stransky N, Greulich H (2011). Inhibitor-sensitive FGFR1 amplification in human non-small cell lung cancer. PLoS One.

[7] Ji W, Yu Y, Li Z, Wang G, Li F, Xia W, Lu S (2016). FGFR1 promotes the stem cell-like phenotype of FGFR1-amplified non-small cell lung cancer cells through the Hedgehog pathway. Oncotarget.

[8] Tan Q, Li F, Wang G, Xia W, Li Z, Niu X, Ji W, Yuan H, Xu Q, Luo Q (2016). Identification of FGF19 as a prognostic marker and potential driver gene of lung squamous cell carcinomas in Chinese smoking patients. Oncotarget.

[9] Mizushima N, Levine B, Cuervo AM, Klionsky DJ (2008). Autophagy fights disease through cellular self-digestion. Nature.

[10] Boya P, Reggiori F, Codogno P. Emerging regulation and functions of autophagy (vol 15, pg 713, 2013). Nat Cell Biol. 2013;15:1017–7. 10.1038/ncb2788 PMC7097732 23817233

[11] Mizushima N, Yoshimori T, Levine B (2010). Methods in mammalian autophagy research. Cell.

[12] Liang XH, Jackson S, Seaman M, Brown K, Kempkes B, Hibshoosh H, Levine B (1999). Induction of autophagy and inhibition of tumorigenesis by beclin 1. Nature.

[13] Qu X, Yu J, Bhagat G, Furuya N, Hibshoosh H, Troxel A, Rosen J, Eskelinen EL, Mizushima N, Ohsumi Y (2003). Promotion of tumorigenesis by heterozygous disruption of the beclin 1 autophagy gene. J Clin Invest.

[14] White E (2012). Deconvoluting the context-dependent role for autophagy in cancer. Nat Rev Cancer.

[15] Strohecker AM, White E (2014). Autophagy promotes BrafV600E-driven lung tumorigenesis by preserving mitochondrial metabolism. Autophagy.

[16] Choi AM, Ryter SW, Levine B (2013). Autophagy in human health and disease. N Engl J Med.

[17] Maiuri MC, Zalckvar E, Kimchi A, Kroemer G (2007). Self-eating and self-killing: crosstalk between autophagy and apoptosis. Nat Rev Mol Cell Biol.

[18] Karpathiou G, Sivridis E, Koukourakis MI, Mikroulis D, Bouros D, Froudarakis ME, Giatromanolaki A (2011). Light-chain 3A autophagic activity and prognostic significance in non-small cell lung carcinomas. Chest.

[19] Inoue D, Suzuki T, Mitsuishi Y, Miki Y, Suzuki S, Sugawara S, Watanabe M, Sakurada A, Endo C, Uruno A (2012). Accumulation of p62/SQSTM1 is associated with poor prognosis in patients with lung adenocarcinoma. Cancer Sci.

[20] Schlafli AM, Adams O, Galvan JA, Gugger M, Savic S, Bubendorf L, Schmid RA, Becker KF, Tschan MP, Langer R, Berezowska S (2016). Prognostic value of the autophagy markers LC3 and p62/SQSTM1 in early-stage non-small cell lung cancer. Oncotarget.

[21] Lin X, Zhang Y, Liu L, McKeehan WL, Shen Y, Song S, Wang F (2011). FRS2alpha is essential for the fibroblast growth factor to regulate the mTOR pathway and autophagy in mouse embryonic fibroblasts. Int J Biol Sci.

[22] Zhang J, Liu J, Huang Y, Chang JY, Liu L, McKeehan WL, Martin JF, Wang F (2012). FRS2alpha-mediated FGF signals suppress premature differentiation of cardiac stem cells through regulating autophagy activity. Circ Res.

[23] Wang X, Qi H, Wang Q, Zhu Y, Wang X, Jin M, Tan Q, Huang Q, Xu W, Li X (2015). FGFR3/fibroblast growth factor receptor 3 inhibits autophagy through decreasing the ATG12-ATG5 conjugate, leading to the delay of cartilage development in achondroplasia. Autophagy.

[24] Cinque L, Forrester A, Bartolomeo R, Svelto M, Venditti R, Montefusco S, Polishchuk E, Nusco E, Rossi A, Medina DL (2015). FGF signalling regulates bone growth through autophagy. Nature.

[25] Gavine PR, Mooney L, Kilgour E, Thomas AP, Al-Kadhimi K, Beck S, Rooney C, Coleman T, Baker D, Mellor MJ (2012). AZD4547: an orally bioavailable, potent, and selective inhibitor of the fibroblast growth factor receptor tyrosine kinase family. Cancer Res.

[26] Klionsky DJ, Abdelmohsen K, Abe A, Abedin MJ, Abeliovich H, Acevedo Arozena A, Adachi H, Adams CM, Adams PD, Adeli K (2016). Guidelines for the use and interpretation of assays for monitoring autophagy (3rd edition). Autophagy.

[27] Biederbick A, Kern HF, Elsasser HP (1995). Monodansylcadaverine (MDC) is a specific in vivo marker for autophagic vacuoles. Eur J Cell Biol.

[28] Tasdemir E, Galluzzi L, Maiuri MC, Criollo A, Vitale I, Hangen E, Modjtahedi N, Kroemer G (2008). Methods for assessing autophagy and autophagic cell death. Methods Mol Biol.

[29] Xie R, Cheng M, Li M, Xiong X, Daadi M, Sapolsky RM, Zhao H (2013). Akt isoforms differentially protect against stroke-induced neuronal injury by regulating mTOR activities. J Cereb Blood Flow Metab.

[30] Boehm JS, Zhao JJ, Yao J, Kim SY, Firestein R, Dunn IF, Sjostrom SK, Garraway LA, Weremowicz S, Richardson AL (2007). Integrative genomic approaches identify IKBKE as a breast cancer oncogene. Cell.

[31] Gao J, Aksoy BA, Dogrusoz U, Dresdner G, Gross B, Sumer SO, Sun Y, Jacobsen A, Sinha R, Larsson E (2013). Integrative analysis of complex cancer genomics and clinical profiles using the cBioPortal. Sci Signal.

[32] Cerami E, Gao J, Dogrusoz U, Gross BE, Sumer SO, Aksoy BA, Jacobsen A, Byrne CJ, Heuer ML, Larsson E (2012). The cBio cancer genomics portal: an open platform for exploring multidimensional cancer genomics data. Cancer Discov.

[33] Anaya J (2016). OncoLnc: linking TCGA survival data to mRNAs, miRNAs, and lncRNAs. PeerJ Computer Science.

[34] Wynes MW, Hinz TK, Gao D, Martini M, Marek LA, Ware KE, Edwards MG, Bohm D, Perner S, Helfrich BA (2014). FGFR1 mRNA and protein expression, not gene copy number, predict FGFR TKI sensitivity across all lung cancer histologies. Clin Cancer Res.

[35] Cancer Genome Atlas Research N (2012). Comprehensive genomic characterization of squamous cell lung cancers. Nature.

[36] Katoh M (2016). Therapeutics Targeting FGF Signaling Network in Human Diseases. Trends Pharmacol Sci.

[37] Gewirtz DA (2014). The four faces of autophagy: implications for cancer therapy. Cancer Res.

[38] Bristol ML, Di X, Beckman MJ, Wilson EN, Henderson SC, Maiti A, Fan Z, Gewirtz DA (2012). Dual functions of autophagy in the response of breast tumor cells to radiation: cytoprotective autophagy with radiation alone and cytotoxic autophagy in radiosensitization by vitamin D 3. Autophagy.

[39] Yousefi S, Perozzo R, Schmid I, Ziemiecki A, Schaffner T, Scapozza L, Brunner T, Simon HU (2006). Calpain-mediated cleavage of Atg5 switches autophagy to apoptosis. Nat Cell Biol.

[40] Kaminskyy VO, Piskunova T, Zborovskaya IB, Tchevkina EM, Zhivotovsky B (2012). Suppression of basal autophagy reduces lung cancer cell proliferation and enhances caspase-dependent and -independent apoptosis by stimulating ROS formation. Autophagy.

[41] Wang Y, Han C, Lu L, Magliato S, Wu T (2013). Hedgehog signaling pathway regulates autophagy in human hepatocellular carcinoma cells. Hepatology.

[42] Wei YJ, Zou ZJ, Becker N, Anderson M, Sumpter R, Xiao GH, Kinch L, Koduru P, Christudass CS, Veltri RW (2013). EGFR-Mediated Beclin 1 Phosphorylation in Autophagy Suppression, Tumor Progression, and Tumor Chemoresistance. Cell.

[43] Li X, Fan Z (2010). The epidermal growth factor receptor antibody cetuximab induces autophagy in cancer cells by downregulating HIF-1alpha and Bcl-2 and activating the beclin 1/hVps34 complex. Cancer Res.

[44] Lipinski MM, Hoffman G, Ng A, Zhou W, Py BF, Hsu E, Liu XX, Eisenberg J, Liu J, Blenis J (2010). A Genome-Wide siRNA Screen Reveals Multiple mTORC1 Independent Signaling Pathways Regulating Autophagy under Normal Nutritional Conditions. Dev Cell.

[45] Chen Y, Xie X, Li X, Wang P, Jing Q, Yue J, Liu Y, Cheng Z, Li J, Song H (2016). FGFR antagonist induces protective autophagy in FGFR1-amplified breast cancer cell. Biochem Biophys Res Commun.

[46] Gorzalczany Y, Gilad Y, Amihai D, Hammel I, Sagi-Eisenberg R, Merimsky O (2011). Combining an EGFR directed tyrosine kinase inhibitor with autophagy-inducing drugs: a beneficial strategy to combat non-small cell lung cancer. Cancer Lett.

[47] Amaravadi RK, Lippincott-Schwartz J, Yin XM, Weiss WA, Takebe N, Timmer W, DiPaola RS, Lotze MT, White E (2011). Principles and current strategies for targeting autophagy for cancer treatment. Clin Cancer Res.

[48] Shinojima N, Yokoyama T, Kondo Y, Kondo S (2007). Roles of the Akt/mTOR/p70S6K and ERK1/2 signaling pathways in curcumin-induced autophagy. Autophagy.

[49] Wang J, Whiteman MW, Lian H, Wang G, Singh A, Huang D, Denmark T (2009). A non-canonical MEK/ERK signaling pathway regulates autophagy via regulating Beclin 1. J Biol Chem.

[50] Ugland H, Naderi S, Brech A, Collas P, Blomhoff HK (2011). cAMP induces autophagy via a novel pathway involving ERK, cyclin E and Beclin 1. Autophagy.

[51] Funderburk SF, Wang QJ, Yue Z (2010). The Beclin 1-VPS34 complex--at the crossroads of autophagy and beyond. Trends Cell Biol.

