# Supplementary figures and images for: FGF2/FGFR1 regulates autophagy in FGFR1-amplified non-small cell lung cancer cells
Source: J Exp Clin Cancer Res. 2017 May 30;36:72. doi: 10.1186/s13046-017-0534-0 (PMC5450166; doi:10.1186/s13046-017-0534-0)

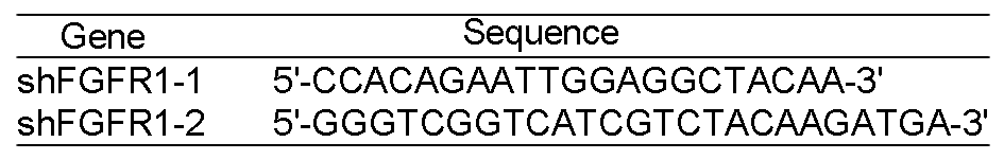

Supplement: Supplementary file 1 — Sequences of FGFR1 shRNA constructs that were used in the study. (TIF 65 kb) [file 13046_2017_534_MOESM1_ESM.tif]

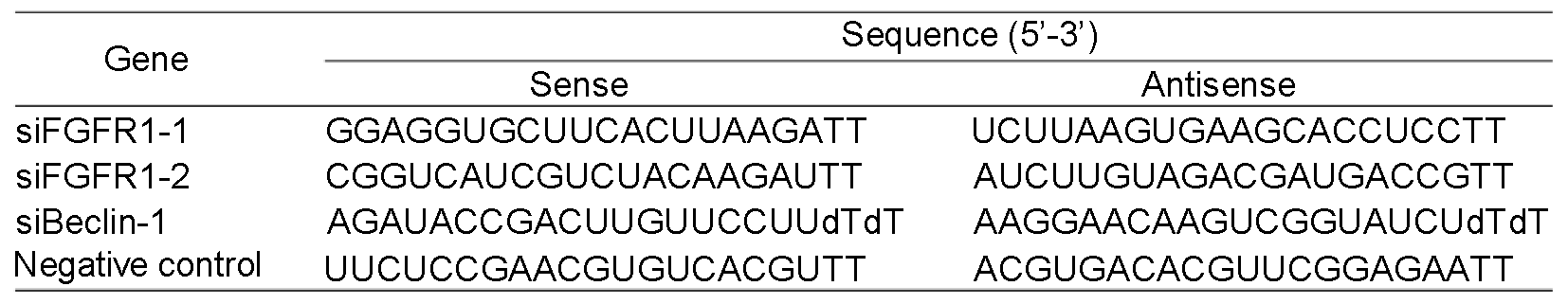

Supplement: Supplementary file 2 — Sequences of all siRNA constructs that were used in the study. (TIF 67 kb) [file 13046_2017_534_MOESM2_ESM.tif]

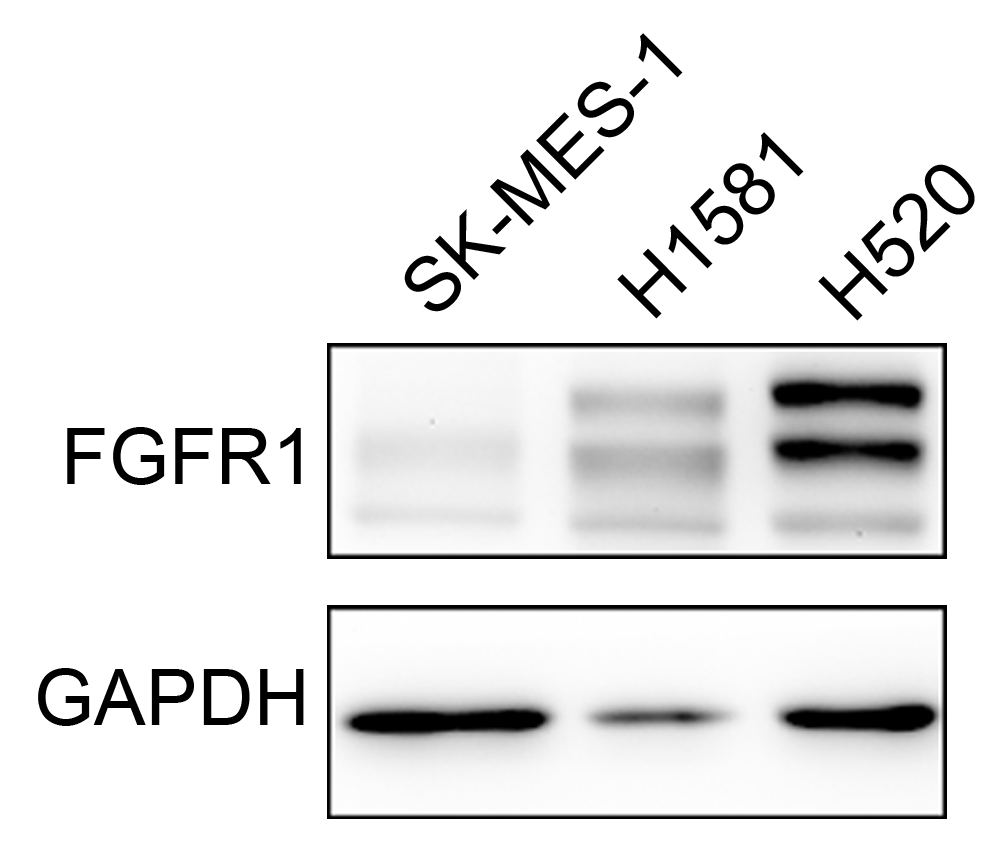

Supplement: Supplementary file 3 — Immunoblot analysis of FGFR1 in NSCLC cell lines. Extracts from NSCLC cells were subjected to immunoblot analysis for FGFR1, GAPDH as a loading control. (TIF 140 kb) [file 13046_2017_534_MOESM3_ESM.tif]

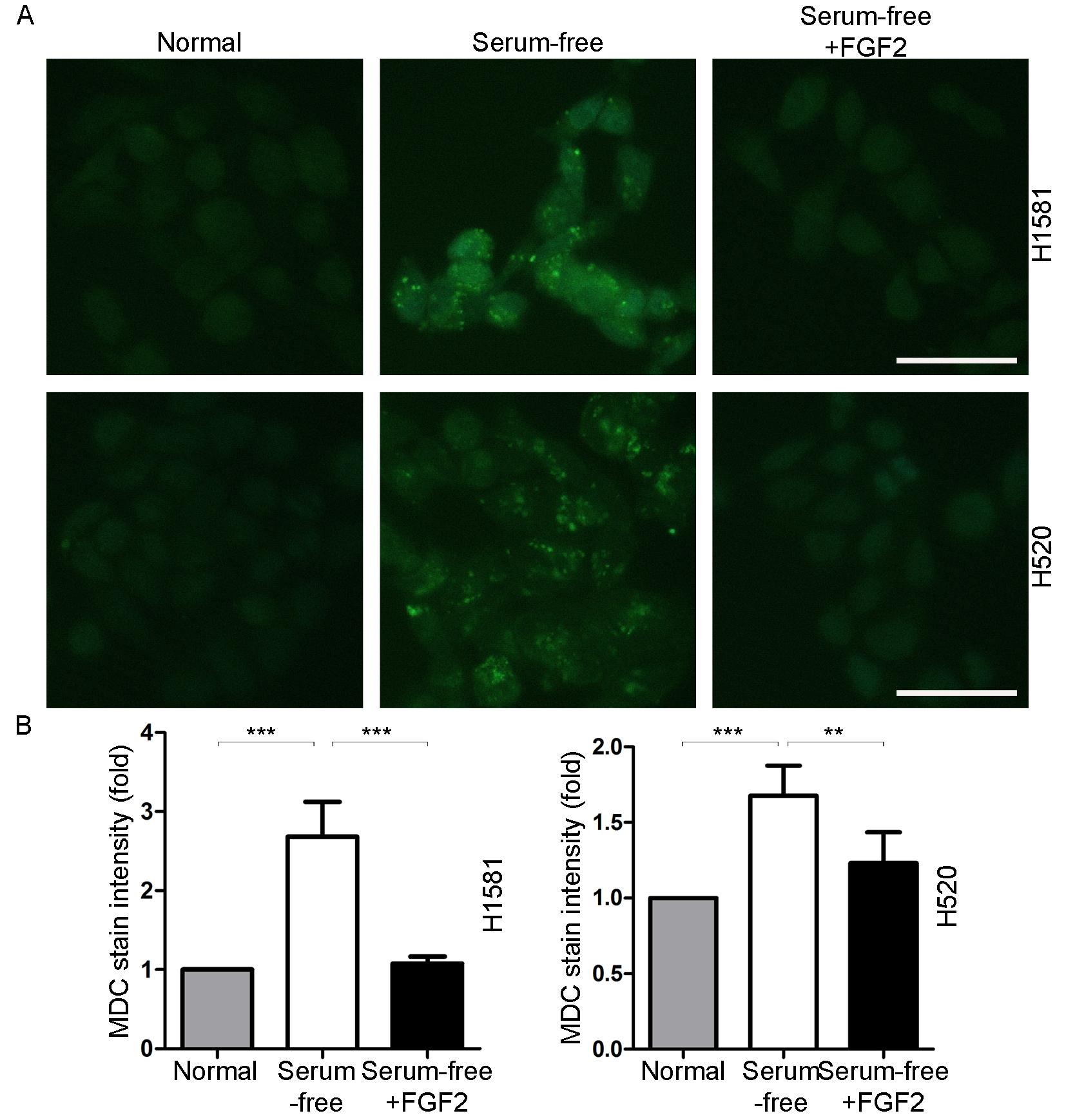

Supplement: Supplementary file 4 — FGFR1 activation inhibits autophagy. (A) MDC staining analysis in H1581 (upper panel) and H520 (lower panel) cells cultured O/N in normal medium, serum-free medium, or serum-free medium plus FGF2 (25 ng/ml, 2 h). Scale bars represent 25 μm. (B) Quantitative results of MDC staining in conditions shown in (A); mean ± SD, n = 3, ***p < 0.001, **p < 0.01. (TIF 2400 kb) [file 13046_2017_534_MOESM4_ESM.tif]

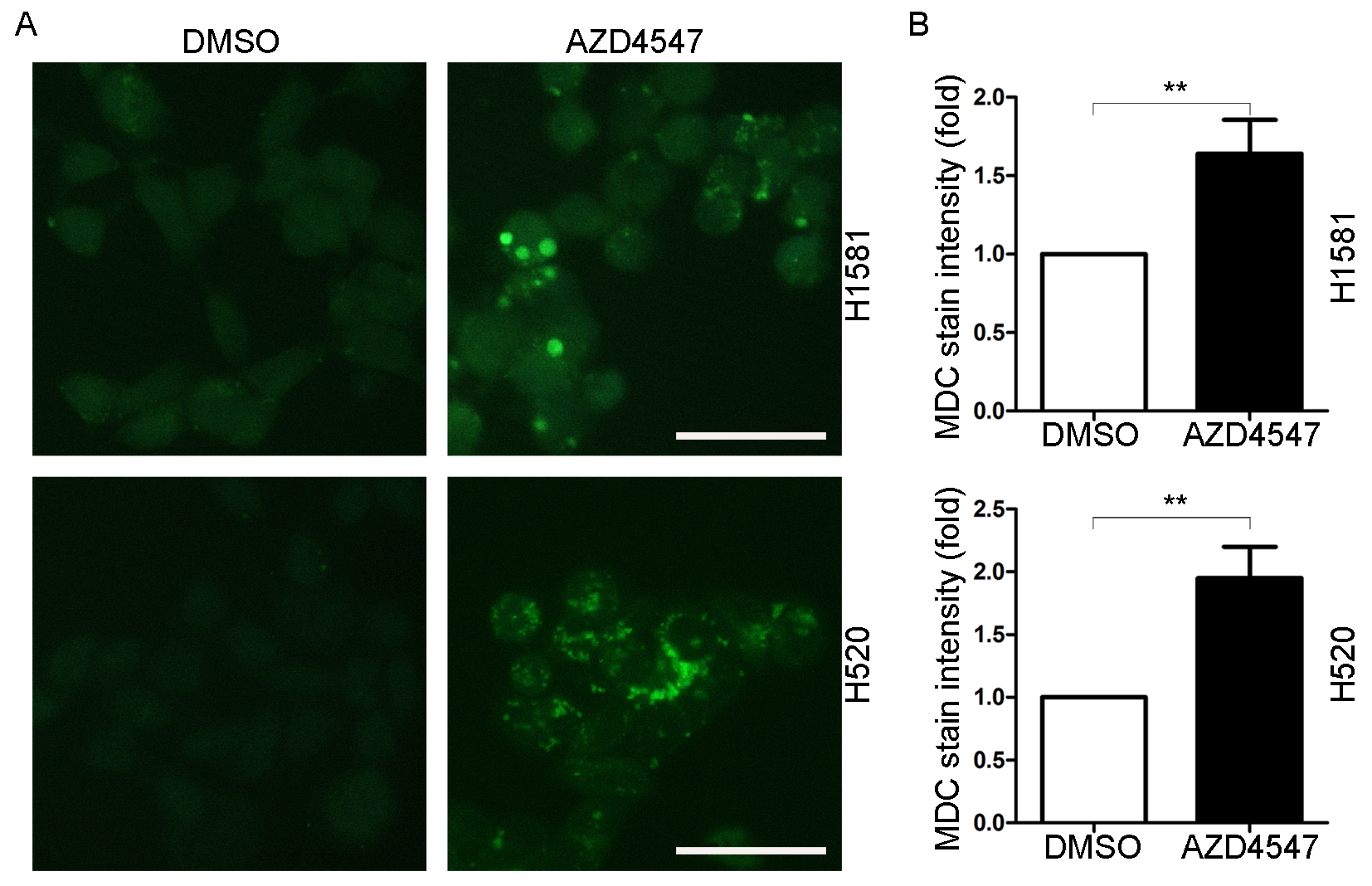

Supplement: Supplementary file 5 — AZD4547 induces autophagy. (A) H1581 and H520 cells grown in 24-well plates were treated with DMSO, or AZD4547 (1 μM) for 24 h. MDC staining was performed and the cells were examined under a fluorescence microscope. Scale bars represent 25 μm. (B) Quantitative results of MDC staining in conditions shown in (A); mean ± SD, n = 3, **p < 0.01. (TIF 1577 kb) [file 13046_2017_534_MOESM5_ESM.tif]

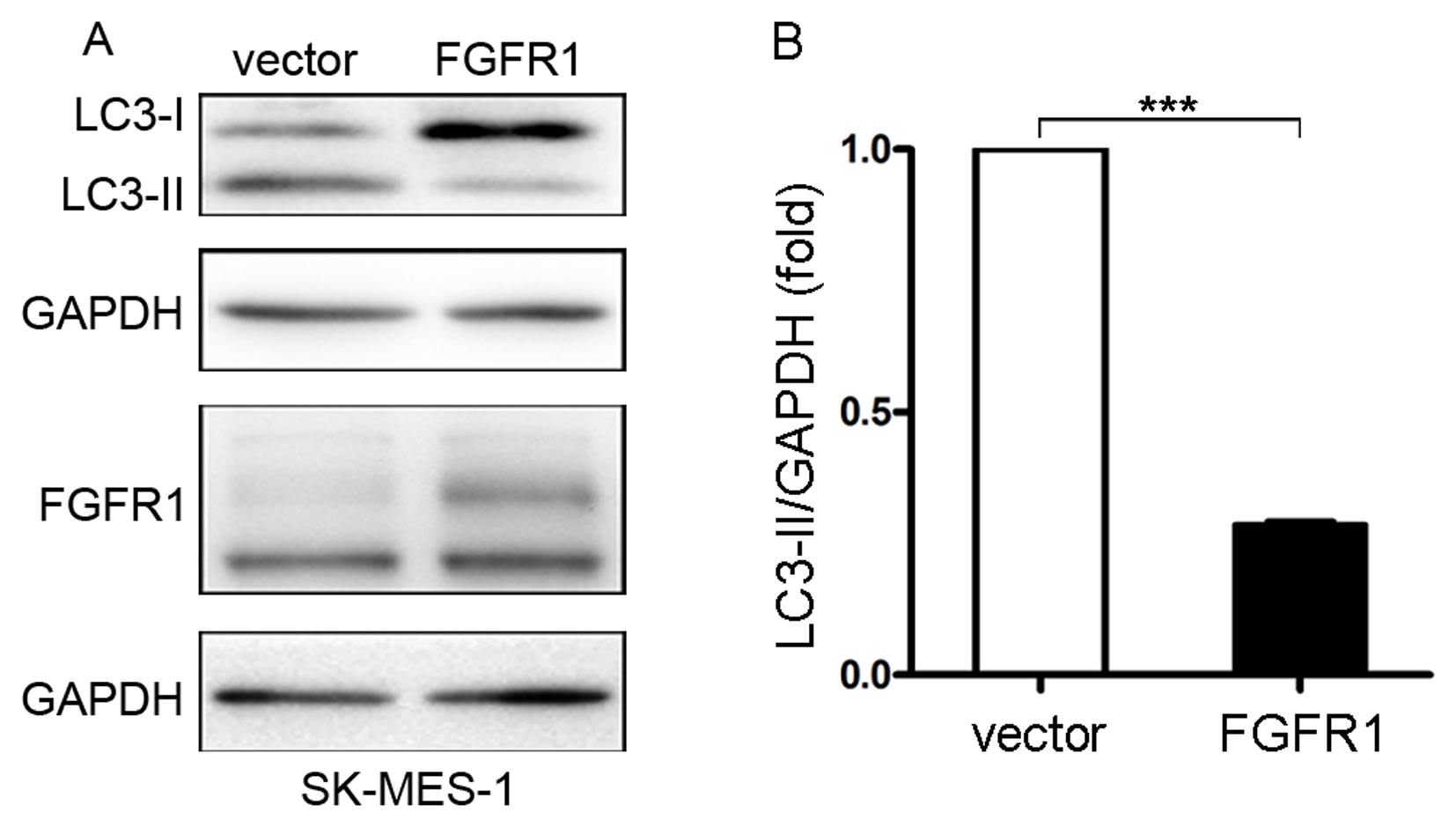

Supplement: Supplementary file 6 — Overexpression of FGFR1 inhibits autophagy in SK-MES-1 cells. (A) The SK-MES-1 cells were transfected with control vector or FGFR1 plasmid. At 48 h post-transfection, LC3-I/II and GAPDH were determined by western blot. The lower panel shows the efficiency of FGFR1 overexpression. (B) Quantification of LC3-II levels in (A); mean ± SD, n = 3, ***p < 0.001. (TIF 321 kb) [file 13046_2017_534_MOESM6_ESM.tif]

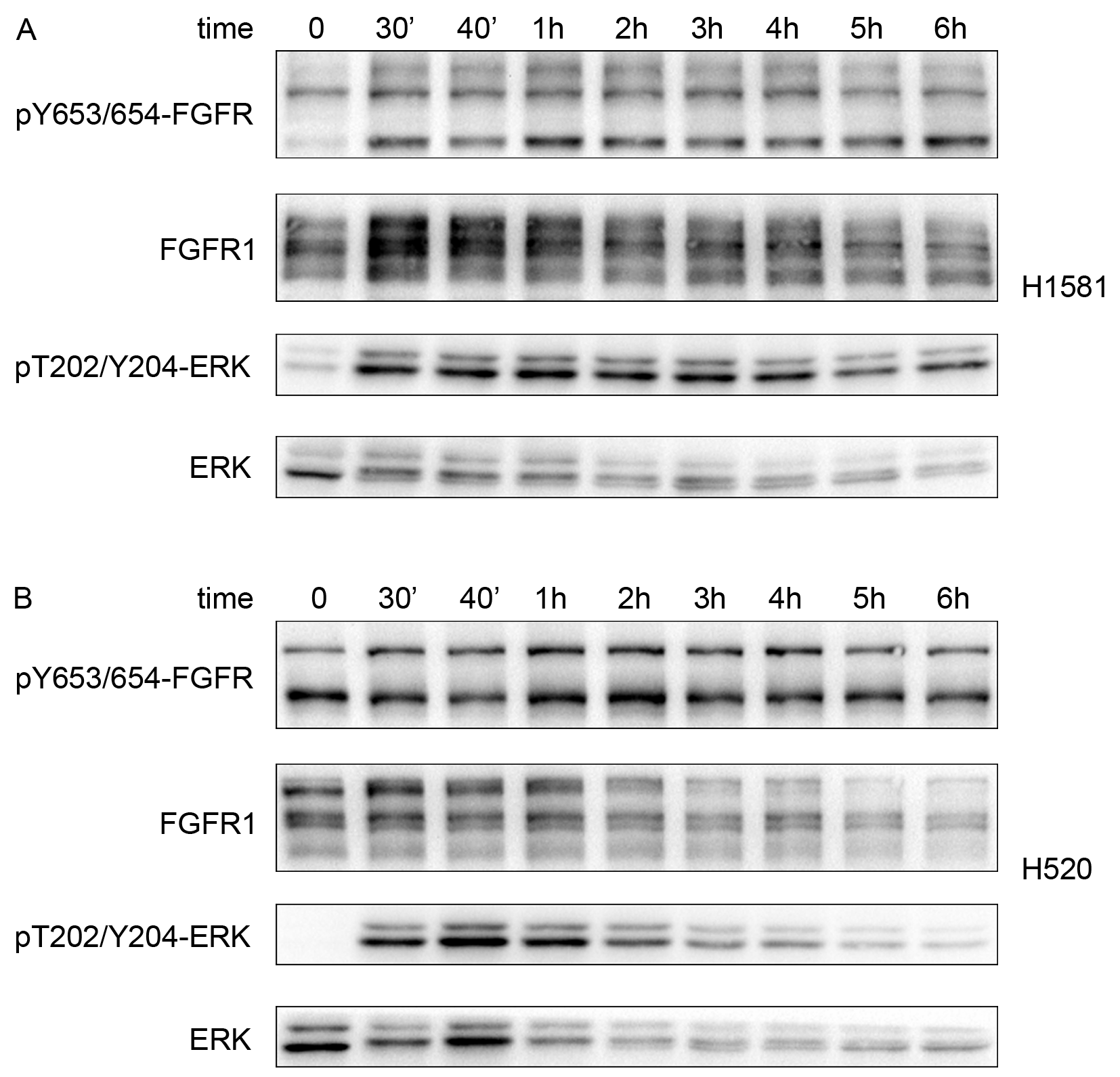

Supplement: Supplementary file 7 — FGF2 activates the ERK/MAPK pathway. (A and B) H1581 and H520 cells treated with 25 ng/ml FGF2 were lysed and probed with the indicated antibodies. The filters were stripped and reprobed for total FGFR1 and ERK to ensure equal loading of cell protein in each lane. (TIF 899 kb) [file 13046_2017_534_MOESM7_ESM.tif]

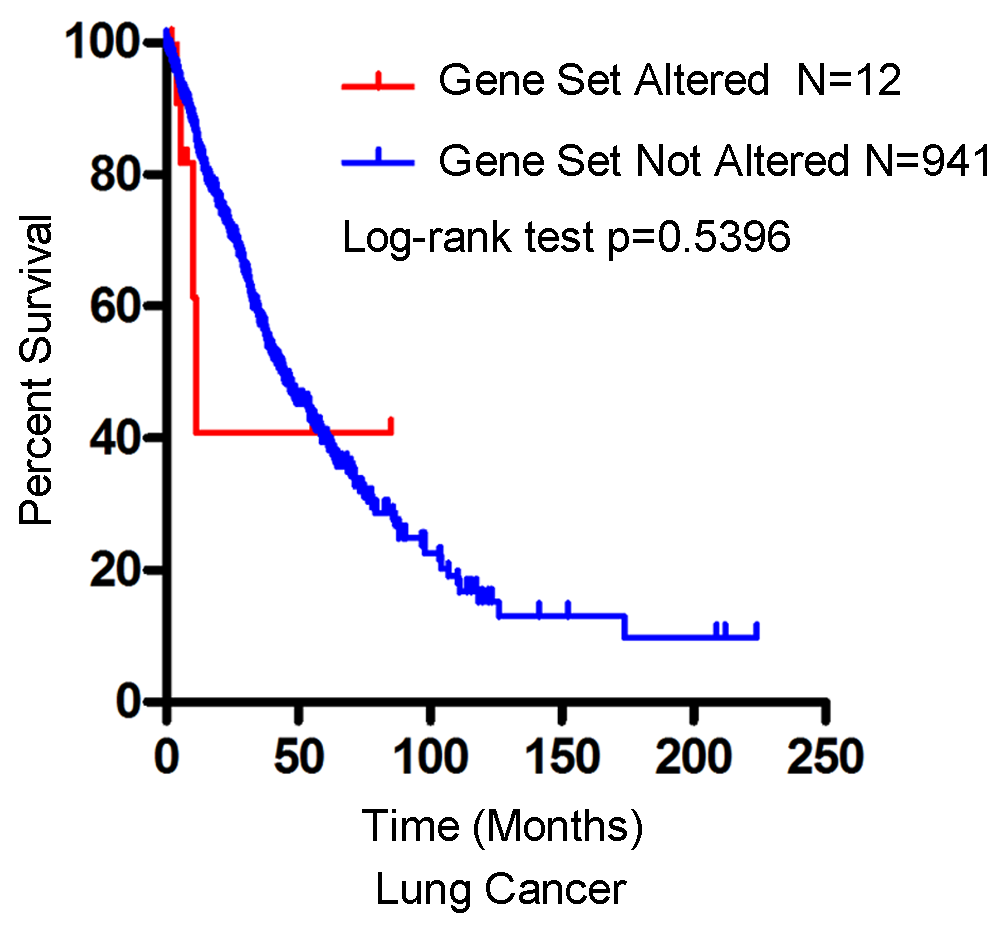

Supplement: Supplementary file 8 — Kaplan-Meier curves for lung cancer patients. OS for patients in TCGA cohort, in lung cancer (N = 12 for cases with alterations in LC3B and N = 941 for cases without alterations in LC3B). P-values are based on the log-rank test. (TIF 179 kb) [file 13046_2017_534_MOESM8_ESM.tif]
